# Enacted Reading Comprehension: Using Bodily Movement to Aid the Comprehension of Abstract Text Content

**DOI:** 10.1371/journal.pone.0169711

**Published:** 2017-01-20

**Authors:** Michael P. Kaschak, Carol M. Connor, Jennifer L. Dombek

**Affiliations:** 1 Department of Psychology, Florida State University, Tallahassee, Florida, United States of America; 2 School of Education, University of California–Irvine, Irvine, California, United States of America; Cardiff University, UNITED KINGDOM

## Abstract

We report a design study that assessed the feasibility of Enacted Reading Comprehension (ERC), an intervention designed to teach 3^rd^ and 4^th^ grade students (n = 40 and 25, respectively) to use gestures to understand an increasingly abstract set of texts. Students were taught to use gestures to understand the idea of “opposing forces” in a concrete setting–the forces at play as tectonic plates move past each other–and then taught to use the gestures to understand opposing forces in more abstract situations. For example, students were taught to use gestures to understand the opposing sides of an argument, and to understand the internal conflicts that arise as individuals are faced with moral dilemmas. The results of our design study suggest that ERC has promise as a method for introducing students to the idea of using gesture to understand text content, and to employ this strategy in a range of reading contexts.

## Introduction

Whether having a conversation with friends, flipping through a magazine to pass the time on an airplane, or poring over a text to study for an exam, the creation of meaning lies at the heart of language use [1, 2]. The centrality of meaning and understanding to the use of language has been an important impetus behind the recent surge of interest in developing approaches and interventions to improve comprehension performance in school-age readers [3]. Compared to the great strides that have been made in improving some aspects of children’s literacy skills (such as the decoding processes needed to translate between orthographic symbols and phonological representations) [4], comparatively little progress has been made in improving comprehension skills [5]. Initiatives such as the Institute for Education Sciences’ Reading for Understanding (RFU) project (which funded the work reported here) have served to highlight the need to understand both the processes involved in reading comprehension, and the mechanisms through which teachers can intervene to support the development of comprehension skills in those students who struggle in this area.

One Reading for Understanding Network team [6] has presented a multi-faceted approach to improving children’s reading comprehension abilities. This approach, which was dubbed *Comprehension Tools for Teachers* (CTT), acknowledges that reading comprehension is a complex process that can fail for a wide range of reasons (e.g., students may not understand narrative structure, or may lack the background knowledge necessary to comprehend a text). CTT therefore presents educators with a range of interventions designed to target comprehension problems in multiple ways (e.g., improving content knowledge, improving syntax use, improving knowledge of text structures, and so on). Implicit in this approach is the idea that there are no single “magic bullet” interventions that will be effective for all students who show signs of comprehension difficulties. Investigators from this team have outlined a "lattice model" of reading development [7] that highlights the extent to which the acquisition of literacy skills is driven by a dynamic interplay of multiple developmental factors. For example, there are reciprocal effects in the development of self-regulation and reading comprehension, such that self-regulation is associated with better comprehension performance, and improved comprehension performance appears to drive subsequent improvements in self-regulation [7].

The purpose of this paper is to present a detailed description of one of the interventions that was outlined in the CTT framework [6]: Enacted Reading Comprehension (ERC). As described below, ERC is an intervention based on *embodied cognition*, or the idea that cognitive processes are grounded in our bodies’ systems of perception and action planning [8, 9]. The intervention is aimed at improving the comprehension of a wide range of texts that involve the abstract notion of opposing forces (e.g., the opposing sides of an argument). Connor et al. [6] present a sketch of ERC, and a report of an efficacy study that looks at the effectiveness of the CTT interventions provides evidence of the value of the ERC intervention (e.g., training on ERC leads to improved scores on the Woodcock-Johnson III Academic Knowledge subtest; [10]). However, neither of these sources provide enough information about ERC, and the initial design work aimed at sharpening the implementation of the intervention, to be useful to readers who wish to assess or build upon these ideas. The current manuscript is intended to fill this gap.

### Enacted Reading Comprehension

The theoretical basis for ERC comes from embodied approaches to language comprehension [11, 12]. A key component to embodied approaches is the idea that linguistic meaning arises through the construction of sensorimotor simulations of the content of the linguistic input [11, 13]. A sentence such as *Meghan served the volleyball to Michael* would be understood in part by simulating the actions involved in serving a volleyball [11], just as a sentence such as *The car approached from the distance* would be understood in part by simulating the visual motion of the oncoming vehicle (e.g., Kaschak et al., 2005). A growing body of behavioral [11, 13, 14] and neuroscientific [15] evidence supports the claim that perceptual and action-based simulations play a role in the comprehension of language. More recently, there has been a wave of interest in applying the principles of embodied cognition to educational settings [16].

One example of an embodied reading intervention is the *Moved by Reading* program developed by Glenberg and colleagues [17, 18]. The idea at the core of *Moved by Reading* is that readers who struggle with comprehension may be experiencing difficulty because they are not devoting enough effort to the construction of a simulation of the text content [17]. The intervention is designed to teach these readers who have weak comprehension to construct simulations. Imagine a student who is being asked to read a story about the events that transpire on a farm. If the student does not have knowledge about farms and farm animals, she may find it difficult to simulate the events that are being described. Similarly, if the student is so focused on the decoding aspect of reading that she does not work to construct a simulation of the text content, she will struggle to understand and recall what she has read. In *Moved by Reading*, the student is presented with a toy farm [17] or an image of the farm on a computer screen [18], and is asked to act out the content of the story (e.g., by moving the cow to the barn). Students are later asked to imagine acting out the story content in an effort to get them to internalize the strategy of constructing a simulation of the text content. A comparison group is asked to re-read the stories rather than acting them out. Glenberg and colleagues [17, 18] subsequently assess reading performance through measures of the students' ability to recall story details, answer comprehension questions, and generate inferences about the texts that were read. Acting out the story content with toys, manipulating images on a computer screen, and imagining the manipulation of toys all result in improvements in reading performance relative to the re-read comparison group.

Similar to *Moved by Reading*, ERC is based on the idea that reading comprehension can be improved by providing readers with strategies for constructing simulations of text content. A major difference between ERC and *Moved by Reading* is the content of the texts that students read. The stories used in *Moved by Reading* are short narratives that describe concrete sequences of events (such as the events that happen on a farm). Such concrete narratives are an obvious case for the application ideas from an embodied approach to comprehension, as the notion of simulation provides a straightforward way to capture the understanding of language about percepts and actions. Indeed, a good deal of the literature supporting the embodied perspective demonstrates that the comprehension of language about concrete situations involves perceptual [19, 20] or motoric [11, 13, 14] simulations. Nonetheless, there is evidence to suggest that abstract concepts may also be understood through sensorimotor simulations. Lakoff and Nunez [21] argue that the understanding of abstract concepts in mathematics is grounded in the concrete understanding of things such as containers. A number of studies also suggest that abstractions such as time [22, 23, 24, 25] and quantity [22, 26, 27] are grounded in a concrete understanding of space and bodily movement through space. More broadly, Lakoff and Johnson [28] outline how the understanding of a range of abstract concepts can be based on our understanding of concrete experience. In the spirit of these accounts, ERC is designed to provide readers with concrete simulation strategies in order to comprehend texts involving more abstract content–specifically, the way that the notion of “opposing forces” can be applied to science texts, persuasive texts, and a novel that features internal conflicts in the main characters.

#### Implementation of ERC

ERC was designed as a small-group (i.e., 4 or fewer students) intervention that would be carried out over a 7-week period. In the parlance of the multi-tiered systems of support or response to intervention, it would be considered a Tier 2 intervention for children who fail to progress adequately when receiving high quality general education Tier 1 instruction. The intervention was carried out 4 days per week over the 7-week period. The small group sessions were done as a pull-out session, where students were taken to a quiet area outside of their classroom. Sessions lasted approximately 20 minutes each day. The 7-week intervention was divided into three phases. Phase 1 involved the reading of expository texts, and lasted 2 weeks. Phase 2 involved the reading of persuasive texts, and lasted 1.5 weeks. Phase 3 involved the reading of a novel, and lasted 3.5 weeks. ERC was developed for students in 3^rd^ and 4^th^ grade.

Phase 1 of the ERC intervention (Expository texts) was designed to introduce students to the idea of *opposing forces* using both concrete and abstract examples. Students were taught to use their hands and arms to construct a physical simulation of the opposing forces involved in these natural events. We chose to use hand and arm movement as a key part of our intervention because gestures are a natural part of language use [29], and language users are accustomed to incorporating gestural content into their understanding of linguistic input [30]. The use of gesture to understand opposing forces was introduced in a more concrete setting (earthquakes–where the opposing forces of the tectonic plates can be understood concretely) and then moved to a more abstract setting (hurricanes and tornadoes–where the opposing forces of the air masses is somewhat less tangible).

Students in both 3^rd^ and 4^th^ grade spent the first week of ERC reading Seymour Simon’s *Earthquakes* [31], and learning about the causes and consequences of earthquakes. The books were read aloud, with the reading alternating between the small group instructor and the students. Students were also introduced to the idea of using hand and arm gestures as a way of understanding the mechanics of an earthquake. As one example, students were asked to press their hands together and slide them back and forth. They were told to pay attention to the friction between their hands as they moved, and the way that their hands could sometimes get “stuck” as they slid past each other. The small group instructor tied these gestures to the idea of tectonic plates sliding past each other, and the way that the plates could get “stuck,” and then release this tension. Students were taught to use their hands to understand faults, and the different kinds of earthquakes that could occur. Throughout, the hand gestures were used to highlight the idea of the opposing forces involved in earthquakes.

Whereas week 1 of the intervention introduced students to opposing forces using a concrete example–the way tectonic plates push on each other as they move, week 2 continued with this theme, but explored opposing forces in a slightly less-tangible context: the movement of air masses. Due to the nature of the reading materials that we had available, it was decided that 3^rd^ grade readers would learn about hurricanes (reading Seymour Simon’s *Hurricanes* [32]) and 4^th^ grade readers would learn about tornadoes (reading Seymour Simon’s *Tornadoes* [33]). Although students in each grade were reading different materials in week 2 of the intervention, the implementation of ERC remained the same. Students were introduced to the idea of air masses pushing against each other, such as the movement that occurs when warm air rises and cooler air moves in to take its place, and the events that occur at weather fronts. They were taught to use hand and arm gestures to depict the forces involved in the development and life of hurricanes (3^rd^ grade) and tornadoes (4^th^ grade). Note that this idea of opposing forces–essentially, air masses pushing against each other–is somewhat abstract compared to the idea of large bodies of rock pushing against each other (as in the lessons about earthquakes).

In Phase 2 of ERC (introduced in the 3^rd^ week of the intervention), students were transitioned away from reading expository texts, and were introduced to the idea of reading a persuasive text (or, a text intended to argue for a particular point of view). Students were reminded about the opposing forces involved in earthquakes, and were reminded of the hand and arm gestures that could be used to represent the forces at work in an earthquake. They were subsequently told that debates and arguments could be thought of in the same way as earthquakes: just as two plates push against each other until a resolution is reached, an argument or debate involves two sides pushing against each other until some resolution is reached. In a persuasive text, the author describes both sides of the argument (i.e., both sets of forces working against each other) before attempting to convince the reader of her argument (the resolution). The students read persuasive texts about grade-appropriate topics (e.g., should school uniforms be required?), and while doing so they 1) discussed the ways that earthquakes and arguments are alike, and 2) filled out a graphic organizer that showed a picture of a fault line, and allowed them to place the elements of each argument on the appropriate side of the fault line (thus making the earthquake/argument analogy explicit).

For Phase 3 of ERC, the final 3.5 weeks of the intervention were spent reading a novel–Linda Sue Park’s *A Single Shard* [34]. We chose this book because it had many good examples of character-internal conflict. For example, the main character of the book, a boy named Tree-Ear, is working for a potter. One night, he sees another potter working on a new technique to inlay designs onto his work. This is a technique that Tree-Ear’s potter lacks. The work of each potter will soon be judged to determine who will be commissioned to work for the Emperor, and so Tree-Ear faces a dilemma–should he steal the idea from this other potter so that his master has a better chance of winning, or should he keep the secret? Students were reminded of the opposing forces of an earthquake, and how that idea could be used to understand persuasive arguments. They were then told that the opposing forces of an earthquake could be used to understand dilemmas of this sort. The students were shown how to use hand and arm gestures to represent the different prongs of the dilemma as they conflicted with each other. The idea of dilemmas-as-opposing-forces was brought up at strategic points as the students read through the novel within their small groups. At these points in the novel, the students also filled out a worksheet that required them to write a few sentences about the nature of the dilemma faced by the characters, and the way that the dilemma was resolved.

The common theme across all three phases of ERC is the idea of opposing forces, and the idea that these forces can be represented through hand and arm gestures. Teaching the students to represent these forces directly, and to do so while they are in the act of reading, provided us with a mechanism for students to ground their understanding of increasingly abstract ideas. We began with earthquakes, where the forces in opposition are the most concrete and the relationship between these forces and the gestures is readily understood. From there we transitioned to understanding the movement of air masses in hurricanes and tornadoes, which introduces the idea that there are times when the opposing forces are things that we cannot directly see. That idea provided the basis for moving toward an understanding of the forces at play in more abstract inter-personal (argument and debate) and intra-personal (moral dilemmas) situations. In tying these different sorts of situation together, it was our hope that students would acquire a strategy for tackling more abstract text content by connecting it to their understanding of more tangible events (e.g., understanding moral dilemmas in terms of the opposing forces, and release of tension, found in earthquakes).

#### Design study

We conducted a design study [35] to facilitate the development of ERC as a small-group intervention. Third and fourth grade students were given content-area pre-tests prior to each phase of the intervention (science texts, persuasive texts, and *A Single Shard*), received the ERC lessons for that phase of the intervention, and then took a content-area post-test. Our primary interest in developing ERC was to develop an intervention for readers with weak reading comprehension skills. As such, we limited participation in this design studies to students who demonstrated standard scores below 100 on the Woodcock Johnson Passage Comprehension scores, where the expected mean is 100 (SD = 15). Due to time constraints (ERC needed to be developed for inclusion in a broader efficacy study; Connor et al., in preparation), we did not have time to run multiple design implementations of our intervention. As such, we ran our design study in two waves that were offset such that the second wave of ERC did not run until after the end of the first phase of the initial wave of ERC. This shortened the amount of time necessary to do two runs of ERC, while also giving us the opportunity to modify the intervention from one wave of the study to the next.

Our design study was aimed at accomplishing two main goals. First, we wished to investigate the extent to which ERC could be implemented in a small-group setting, and that ERC could be implemented within the timeline that we developed. Due to time constraints, we did not do formal data collection about the feasibility of the intervention. Rather, we relied on reports from our interventionists during design team meetings about whether there were any difficulties in implementing ERC as intended in the time allotted. Our interventionists reported only minor difficulties in carrying out ERC as intended during the first round and then implemented revised lesson plans that resolved them for the second round.

Our second goal was to take an initial look at whether students can learn the relevant content information within the context of ERC. That is, we sought to demonstrate that the addition of gestures and other components of ERC would not distract the students from acquiring information from the texts they were reading. Because this was a design study, it was beyond the scope of our project to collect data from a control group for comparison purposes. It was likewise beyond the scope of our project to collect data to assess which component(s) of ERC were responsible for driving any learning that we observed. Instead, we had the more modest aim of using performance on our pre- and post-test items to demonstrate that students could learn the text-relevant material within the context of the ERC intervention.

## Method

### Participants

We recruited 127 students (74 3^rd^ grade students, and 53 4^th^ grade students) to participate in our study. The students attended a Title 1 school in North Florida where 77.8% of the students qualified for free or reduced price lunch. We received written parental consent for participation for 85 students (52 3^rd^ grade, 33 4^th^ grade). [Note: we received parental consent from an additional 17 students, but the consent forms were received well after the deadline for participation in the study. Thus, these students were not tested]. The racial and ethnic breakdown of the consented student sample was 82% Black or African American, 12% white, 4% Hispanic and 2% Asian or Multiracial. All students were given the Woodcock Johnson Passage Comprehension assessment (along with other standardized assessments; see below). Students with standard scores above 100 on the Passage Comprehension assessment were excluded from the intervention. Based on this criterion, the final sample included 40 third grade students (15 in the first wave of ERC, 25 in the second wave of ERC) and 25 fourth grade students (12 in the first wave of ERC, 13 in the second wave of ERC). [Note: An additional 3 students in 4^th^ grade qualified for ERC, but were excluded from the final sample due to excessive absences. Two of these students completed the Phase 1 pre-tests, but did not complete the post-tests and did not complete any tests for Phases 2 and 3. The remaining student completed the pre- and post-tests in Phase 1, but did not complete any tests in Phases 2 and 3]. Representation of male and female students was almost even in both the final and consented sample. The racial and ethnic breakdown of the final sample was 81% Black or African American, 7% White, 3% Hispanic and 3% Asian or Multiracial. In the final sample, four of the third grade students and one of the fourth grade students were designated as students with language impairment, one third grade student was a child with autism and one third grade student was a child with speech impairment.

### Design Team

The design team included three Ph.D.-level scientists, one with clinical training in speech-language pathology and doctoral work in developmental psychology and special education (with an emphasis on language, literacy, and culture), one with a Ph.D. in cognitive science (with an emphasis on language and embodied cognition), and one with a Ph.D. in Reading and Language Arts. In addition, two certified elementary school teachers, of which one was pursuing a PhD. in Reading and Language Arts, were included in the design team and also implemented lessons with several of the small groups.

### Intervention Teachers

The intervention teachers included two members of the design team and three additional certified teachers. The intervention teachers received three half-day training sessions aligned with the three phases of the study (expository texts, persuasive texts, and narrative texts) and an additional half-day training session focused on behavior management, administering pre- and post-tests, and communicating with classroom teachers. The intervention teachers who were on the design team gathered feedback from all of the other interventionists, and presented this information during our regular project meetings so that adjustments to the implementation of the project could be made. We did not conduct formal fidelity checks for our interventionists’ implementation of the lessons.

### Materials

The study involved the use of four books: Seymour Simon's *Earthquakes* (31), *Tornadoes* (33), and *Hurricanes* (32), and Park's *A Single Shard* (35). There were also researcher-designed materials: the pre-test and post-test measures, the lesson plans given to interventionists, and the graphic organizer used along with the persuasive texts. The materials and data from this study are available for download on the Open Science Framework (https://osf.io/xhbx4/).

We constructed content area pre- and post-tests for each of the three phases of the study. The earthquakes pre-test included 11 content-area items, and 2 control items assessing knowledge of atoms. The post-test contained the same 13 items, plus an additional 2 items assessing knowledge of volcanoes. The hurricanes pre-test contained 11 content-area items, and 2 control items assessing knowledge of chemistry. The post-test contained the same 13 items, plus an additional 2 items assessing knowledge of tornadoes. For the second wave of ERC, the post-test also contained one additional content-area question. The tornadoes pre-test contained 10 content-area items, and 2 control items assessing knowledge of chemistry. The post-test contained the same 12 items, plus an additional 2 items assessing knowledge of hurricanes. The additional post-test items were added in an effort to assess transfer of knowledge (e.g., does learning about hurricanes inform you about tornadoes?). Because we did not include these items on the pre-test, and because the transfer issues are somewhat removed from the main purpose of this report, we excluded these items from the analyses reported below. The pre-test for persuasive texts was a set of questions that a) asked students to read and respond to a short persuasive text, and b) asked students about their understanding of persuasion and argumentation. There were 8 content-area items and 2 control items assessing knowledge about animals. The post-test for persuasive texts contained the same 10 items. All of these tests were multiple-choice tests. Finally, the pre- and post-test for *A Single Shard* contained 10 short-response questions that asked students about the plot elements of the novel, and about their understanding of conflict and dilemmas. These short responses were scored as 1 for an answer that was completely correct, .5 for items that were partially correct, and 0 for items that were incorrect. The scoring was done by several raters. The raters were trained on a subset of the students’ responses and training continued until each rater achieved a kappa of .70. Once this goal was achieved, the entire set of responses was scored, with the responses split among the individual raters. When the scoring was complete, a subset of 20% of the responses (split across all raters) was scored by a second rater. In cases where this resulted in a kappa < .70, the items were re-scored until this standard was met. Once the final scoring was complete, one additional rater scored all of the responses. This rater's scoring agreed with the final scoring on 93% of the student responses, indicating that the scoring was reliable.

We also administered the Letter-Word Identification, Picture Vocabulary, Passage Comprehension, and Oral Comprehension subtests of the Woodcock-Johnson-III Tests of Achievement (WJ-III; [36]) to participating students (Note: these data were collected over the 2011–2012 school year, prior to the availability of the Woodcock-Johnson IV in 2014). All three measures have reliabilities between .77 and .98 according to the technical manual. These measures were administered prior to the beginning of the design study. Although our primary interest was in administering the Passage Comprehension measure to find students on the lower end of the range of comprehension performance in these classrooms, we administered the other measures so that 1) we would have a fuller picture of these students' language-related skills, and 2) we could later examine the relationship between these measures and test performance on ERC in an exploratory fashion, if we desired. We have not yet explored this avenue, but we nonetheless report data on all of our measures so that the reader has a full picture of the students in our sample.

### Procedure

Data collection on this project was approved by the Institutional Review Board of Florida State University. After receiving permission from the relevant school board, principal, and teachers, we distributed consent forms to the parents of the 3^rd^ and 4^th^ grade students in our targeted school. Students whose parents gave written consent to participate in the study were given the Woodcock-Johnson assessments described above so that we could assess their reading and language skills. We excluded students with standard scores on the Woodcock Johnson Passage Comprehension assessment greater than 100 at this point. The remaining students were assigned to be in a small group either in the first or second wave of the intervention.

The intervention proper was carried out over 7 weeks. Students were pulled out of their normal classroom in groups of 4. A small-group interventionist conducted each of the ERC lessons. We unfortunately lost data tracking absences from our small group sessions for some of the participating children. We have data for 31 of the 40 3^rd^ grade children who participated. The average number of absences for these children over the course of the 28 days of the intervention is 1 (*SD* = 2.04). We have data for 17 of the 25 4^th^ grade children who participated. The average number of absences over the course of the intervention is 1.5 (*SD* = 3.14). Although incomplete, these data suggest that attendance rates for the intervention were quite high.

The ERC lessons themselves involved a mix of discussion (where the interventionist introduced topics, or recapped previous lessons), open-ended prompts (where the interventionist elicited responses and gestures from the students), and reading. The interventionist did some of the reading, and students were asked to take turns doing some of the reading. Due to the length of *A Single Shard*, students were provided with copies of the book and asked to complete some of the reading on their own outside of the group sessions. The amount of outside reading done varied from group to group, and depended on both the amount of reading that was completed in the group session, and the amount of reading required to prepare the students for the next session. The timeline for the first wave of the study is presented in Table 1. The second wave of the study commenced a week after the first phase of the first wave ended (i.e., in week 4), and followed the same timeline as the first wave of the study. This allowed us to have time to assess the outcome of Phase 1 from the first wave based on our observations of the lessons and interventionist feedback, and implement any changes necessary for the second wave.

**Table 1:** Study timeline.

Phase	Week(s)	Description
**1**	1	Pre-test on earthquakes
		Pre-test on hurricanes (3^rd^ grade) or pre-test on tornadoes (4^th^ grade)
		ERC lessons on earthquakes
	2	ERC lessons on hurricanes (3^rd^ grade) or tornadoes (4^th^ grade)
		Post-test on earthquakes
		Post-test on hurricanes (3^rd^ grade) or post-test on tornadoes (4^th^ grade)
**2**	3	Pre-test persuasive texts
		ERC lessons on persuasive text
	4	Post-test persuasive texts [Second wave of ERC begins here]
**3**	4	Pre-test narrative text
		ERC lessons on narrative text
	5–6	ERC lessons on narrative text
	*7*	ERC lessons on narrative text
		Post-test narrative text

*Note*. Pre and post-tests and lessons are the same for both 3^rd^ and 4^th^ grade unless noted.

The implementation changes made between the first and second waves of ERC were as follows. For the science-based lessons changes included incorporating time each day to review the hand motions taught during the prior lesson(s), and allotted the students time to preview the text at the start of each lesson. Reviewing the hand motions allowed the interventionists to determine what the students were able to recall from the prior lesson(s) and emphasize these aspects as needed throughout the lesson ensure understanding. The text previewing allowed the students time to look at the engaging photographs and diagrams related to the natural disasters prior to reading, which allowed them to focus on the text once reading began. For the persuasive lessons, additional instruction regarding the use of a graphic organizer was added. For example, interventionists would explain to students that when competing the graphic organizer the use of full sentences was not necessary, as well, only critical (relevant) content needed to be included and did not need to be written verbatim. For the narrative lessons, a list of vocabulary words were added to each lesson plan. The list of vocabulary words was selected based on complexity and/or specialization to the content of the narrative text, both of which made comprehension difficult for students at times. The list served as a reminder for the interventionists to stop when a vocabulary word was approached during the reading and have a short conversation with the students about its meaning in the context of the story. In addition, the pacing of the lessons was fine-tuned such that a) each daily lesson plan dictated the amount of text that should be covered in a given day, and b) the plans dictated the exact questions students should be asked at various parts of each chapter. These changes ensured that the interventionists could give the more challenging chapters or concepts in the text sufficient attention.

## Results

### Descriptive Statistics

The results of our Woodcock-Johnson pre-tests are presented in Table 2. For completeness, the table reports the descriptive statistics for all Woodcock-Johnson tests for our full pre-test sample, and for the group of students who were eligible to participate in the intervention. Of the subtests administered, the Letter-Word Identification standard scores were collectively the highest across grades and samples with means ranging from 103.25 to 105.23; this subtest required students to read words of increasing difficulty in a list format. Across grades and samples, the Passage Comprehension standard scores were collectively the lowest with means ranging 91.52 to 95.23; this subtest required students to supply the missing word for sentences and later paragraphs of increasing difficulty. In both grades, the Passage Comprehension subtest shows the largest standard score difference between the full pre-test sample and the sample who was eligible for participation. This is because the Passage Comprehension subtest was used to determine which students were eligible for the intervention.

**Table 2:** Pretest Standard Score Means for Woodcock-Johnson Subtests by Grade and Sample.

	Grade 3		Grade 4	
	Full Sample	Eligible Sample	Full Sample	Eligible Sample
*N*	52	40	33	25
Letter-Word Identification SS (SD)	105.23 (7.93)	103.25 (7.05)	104.40 (7.66)	104.36 (7.52)
Picture Vocabulary SS (SD)	93.65 (7.06)	92.23 (6.50)	95.82 (8.27)	96.52 (8.73)
Passage Comprehension SS (SD)	95.23 (7.39)	92.15 (5.13)	93.85 (7.80)	91.52 (5.81)
Oral Comprehension SS (SD)	99.73 (10.27)	98.78 (10.08)	98.70 (12.01)	99.96 (12.64)

*Note*. SS is Standard score; SD is Standard Deviation.

### Test Scores

The pre- and post-test percentage correct for each topic (Earthquakes, Hurricanes, Persuasive texts, and *A Single Shard* for third grade, and Earthquakes, Tornadoes, Persuasive texts, and *A Single Shard* for fourth grade) was calculated for each participant. The means and standard deviations for these conditions in each grade are presented in Tables 3 and 4. These scores were analyzed using a 2 (Time: pre-test vs. post-test) x 2 (Item type: content area vs. control) ANOVA with repeated measures on both factors (note that for *A Single Shard*, we used a one-way repeated measures ANOVA to assess the content area items, as there were no control items on the tests). We followed up the factorial ANOVAs by analyzing the simple main effects of Time for each item type (content area or control) within each content area. The results of these ANOVA and simple main effects tests are presented in Tables 5 and 6. We included Cohen’s *d* for each of the simple main effects tests to provide an index of the magnitude of change from the pre-test to the post-test. [Note: There were a few instances where participants who were ineligible for ERC were mistakenly given pre-tests or post-tests for a particular content area (typically during the first week of the intervention). Because the participants were ineligible for ERC, these observations were not included in our analysis of the test items].

**Table 3:** Mean Proportion Correct for Grade 3 Tests (Standard Deviation in Parentheses).

	Pre-test	Post-test
**Earthquakes**		
Content items	.28 (.15)	.50 (.19)
Control items	.23 (.30)	.23 (.23)
**Hurricanes**		
Content items	.23 (.10)	.35 (.18)
Control items	.13 (.25)	.17 (.26)
**Persuasive Texts**		
Content items	.38 (.21)	.48 (.23)
Control items	.28 (.28)	.28 (.34)
**A Single Shard**		
Content items	.02 (.06)	.24 (.26)

**Table 4:** Mean Proportion Correct for Grade 4 Tests (Standard Error in Parentheses).

	Pre-test	Post-test
**Earthquakes**		
Content items	.44 (.17)	.66 (.18)
Control items	.44 (.33)	.40 (.38)
**Tornadoes**		
Content items	.29 (.17)	.56 (.18)
Control items	.23 (.29)	.44 (.37)
**Persuasive Texts**		
Content items	.59 (.25)	.68 (.23)
Control items	.54 (.32)	.52 (.34)
**A Single Shard**		
Content items	.06 (.07)	.42 (.24)

**Table 5:** ANOVA Results for 3^rd^ Grade Test Scores.

	*df*	*F*	*p*	*d*
**Earthquakes**				
Time	1, 39	8.07	< .01	
Item Type	1, 39	17.42	< .01	
Time x Item Type	1, 39	11.6	< .01	
Simple Main Effects for Item Types across Time				
Content items	1, 39	41.24	< .01	1.03
Control items	1, 39	< 1	0.84	0
**Hurricanes**				
Time	1, 38	6.25	0.02	
Item Type	1, 38	16.00	< .01	
Time x Item Type	1, 38	1.57	0.22	
Simple Main Effects for Item Types across Time				
Content items	1, 38	15.19	< .01	.72
Control items	1, 38	< 1	0.47	.12
**Persuasive Texts**				
Time	1, 39	1.35	0.25	
Item Type	1, 39	12.54	< .01	
Time x Item Type	1, 39	2.73	0.11	
Simple Main Effects for Item Types across Time				
Content items	1, 39	5.61	0.02	.39
Control items	1, 39	< 1	1	0
**A Single Shard**				
Time	1, 39	24.80	< .01	.94

*Note*. One participant missed a test session for the Hurricanes unit, and was excluded from that analysis.

**Table 6:** ANOVA Results for 4^th^ Grade Test Scores.

	*df*	*F*	*p*	*D*
**Earthquakes**				
Time	1, 24	3.24	0.09	
Item Type	1, 24	6.26	0.02	
Time x Item Type	1, 24	7.33	0.01	
Simple Main Effects for Item Types across Time				
Content items	1, 24	32.43	< .01	1.12
Control items	1, 24	< 1	0.66	-.09
**Tornadoes**				
Time	1, 23	19.38	< .01	
Item Type	1, 23	2.72	0.11	
Time x Item Type	1, 23	< 1	0.55	
Simple Main Effects for Item Types across Time				
Content items	1, 23	23.03	< .01	1.13
Control items	1, 23	4.83	0.04	.46
**Persuasive Texts**				
Time	1, 24	< 1	0.34	
Item Type	1, 24	3.03	0.1	
Time x Item Type	1, 24	3.79	0.06	
Simple Main Effects for Item Types across Time				
Content items	1, 24	6.34	0.02	.51
Control items	1, 24	< 1	0.71	-.08
**A Single Shard**				
Time	1, 24	52.39	< .01	1.72

Note: One student missed a test session for the Tornadoes unit, and was excluded from that analysis.

We sought to demonstrate that students can learn in the context of ERC in two ways. First, the 2 x 2 ANOVAs were expected to show a Time x Item type interaction, such that performance on the content area questions improved from pre-test to post-test, and performance on the control items did not. Second, the simple main effects tests were expected to show an improvement in scores from pre-test to post-test for the content area items, but no improvement in scores for the control items. The results of our analyses provided partial support for our predictions. In both third and fourth grade, there was a significant Time x Item type interaction for the Earthquakes unit (*F*(1, 39) = 11.60, *p* < .01 for 3^rd^ grade, and *F*(1, 24) = 7.33, *p* = .01 for 4^th^ grade). The interaction failed to reach significance for any of the other content areas, despite the fact that each content area showed the expected pattern of results (larger pre- to post-test gains for content area items than for control items). The statistical weakness of these results may be a function of a relative lack of statistical power in our design–the sample sizes are comparatively small (n = 25 for 4^th^ grade, and n = 40 for 3^rd^ grade).

The results of the simple main effects tests provide stronger support for our predictions. As seen in Tables 5 and 6, the pre-test to post-test difference was statistically significant for the content area questions in each area that was assessed. This was true for both grades. The pre-test to post-test differences for the control items was not significant for any content areas except for Tornadoes in 4^th^ grade, *F*(1, 23) = 4.83, p = .04. There is no apparent explanation for the improvement on the control items in this condition, so this result may be a statistical fluke. Overall, the results thus provide support for the claim that students acquired content area knowledge as a result of their participation in ERC, and that ERC did not result in changes in content area knowledge that were not part of the intervention (i.e., the control content areas). The Cohen's *d* values for the simple main effects on the content area questions show that the pre-test to post-test changes were generally positive and medium-to-large in magnitude.

## Discussion

We embarked upon this project to demonstrate that principles of embodied cognition that had been applied in other reading interventions (17) could be used as the basis for an intervention aimed at a range of different types of text: science texts, persuasive texts, and long narratives. ERC was designed to use gestural content to teach students about the concept of "opposing forces," a concept that could be used to teach readers about a number of different domains: the movement of tectonic plates and weather masses, argument and debate, and internal conflict. We implemented ERC as a 7-week, small reading group intervention that was performed by trained interventionists outside of the context of the students' classroom. There are some noteworthy results from our project.

First, it appears that ERC is a feasible intervention to employ in the context of science texts, persuasive texts, and narrative texts. Our interventionists reported little difficulty in implementing the intervention in small groups, and it was reasonable to complete the entire intervention within a 7-week period. Although it is certainly feasible to do ERC in 7 weeks, our interventionists indicate that it might be prudent to extend the narrative text component of the intervention with some children. We implemented ERC by asking students to do some reading of the book on their own. To the extent that there is variability in how much an individual student will follow the directive to read the book on their own, interventionists may make better progress with this component of ERC by doing more reading in the small group setting, and relying less on the students to read outside the group.

A second noteworthy result from our project is that students who participate in ERC appear to learn at least some of the relevant content for each component of the intervention (as demonstrated by our simple main effects tests). It is interesting to note that the only content area to show a significant Time x Item type interaction was Earthquakes. The lessons on Earthquakes are the ones that discuss opposing forces in the most concrete, tangible way—rocks pushing against each other–and this maps most directly onto the concrete, tangible actions that students were asked to perform. It might therefore be expected that the gestures employed in ERC would have their strongest effect on performance in the Earthquakes domain. Whether this "concreteness" effect is genuine, or whether it reflects something less interesting (e.g., Earthquakes is the first lesson, and the gestural component of ERC is more novel to the students at this point in the intervention), is a question that will need to be resolved in future research. As we consider these results, it is also worth pointing out that the post-test scores appear to be somewhat low (between .24 and .68 correct). The low scores may reflect the learning dynamics of our study, as students were limited in their study time for the target texts, and were given the post-tests without the opportunity to prepare outside of the small group sessions. Despite the fact that the post-test scores were a bit on the low side, the fact that the pre-test to post-test effect sizes for the content areas that we assessed were generally in the medium-to-large range suggests that, at the very least, the addition of the gestures and other elements of ERC does not distract students from acquiring the relevant targeted knowledge.

Although we consider this design study to be a success in demonstrating initial promise for ERC as a reading intervention, there are some weaknesses to the study that should be acknowledged. As noted earlier, we did not have a formal procedure of collecting and analyzing feedback about the feasibility of ERC from our interventionists outside of design team meetings. A number of the interventionists were on our design team, and the verbal reports we received from them as the project unfolded indicated that the intervention is doable within the constraints described here. Nonetheless, the lack of formal, detailed feedback is a shortcoming of our project. We also did not assess the reliability of our researcher-designed assessments. Although the assessments were based closely on the study material (and therefore serve as a reasonable test of the students' knowledge), more work can be done to validate the measures.

A broader weakness of this work is that we did not include a genuine control group (e.g., a "business as usual" control group to learn about each content area) in our study. Although we are using our pre- to post-test gains in performance as evidence that students can learn about content areas through ERC, we do not have evidence that students learn more through ERC than they might through a more traditional classroom setting or a different set of interventions. Note, however, that a comparative efficacy study involving ERC has shown that training on this intervention can impact more distal measures of scholastic achievement (in this case, the Woodcock-Johnson Academic Knowledge subtest; 10).

A final shortcoming of the current project is that the design of our study did not allow us to assess the "active ingredient" that may be driving student performance. In addition to the gestural component of ERC, we included graphic organizers, vocabulary lists, and other supports in an effort to make the project more doable with the students. Thus, although it is our expectation that the gestural component of ERC aids in the comprehension process, further work will be necessary not only to determine the efficacy of ERC relative to various control groups, but also to determine the extent to which the "embodied" components of the intervention are key to the success of the intervention.

The results of this design study suggest that ERC is a feasible small-group intervention that can be applied to the reading of a range of different text types. Though we are limited in the conclusions that can be drawn about the efficacy of ERC at this point, we nonetheless feel that we have shown that it is possible to apply the principles of embodied cognition to a literacy intervention that encompasses texts that present readers with a range of content-area and conceptual demands.
